# Primary Congenital Glaucoma with Delayed Suprachoroidal Hemorrhage following Combined Trabeculotomy Trabeculectomy and 5-Fluorouracil

**DOI:** 10.1155/2015/163859

**Published:** 2015-12-24

**Authors:** Roseline Duke, Anthonia Ikpeme

**Affiliations:** ^1^Department of Ophthalmology, University of Calabar Teaching Hospital, Calabar, Cross River State, Nigeria; ^2^Department of Radiology, University of Calabar Teaching Hospital, Calabar, Cross River State, Nigeria

## Abstract

*Background*. Delayed postoperative suprachoroidal hemorrhage (DSCH) may occur following intraocular surgery for the treatment of glaucoma. It is considered to be a rare and debilitating event if not managed appropriately. Reported herewith is a case of Primary Congenital Glaucoma followed by DSCH with successful immediate surgical intervention and visual restoration.* Patient and Method*. An 8-month-old male child had bilateral Primary Congenital Glaucoma (PCG). Combined Trabeculotomy Trabeculectomy with 5-Fluorouracil (5FU) was performed. He developed delayed suprachoroidal hemorrhage (DSCH) within 24 hours after intraocular surgery which was drained. In addition, he developed exposure keratopathy and left amblyopia.* Outcome*. Resolution of the DSCH was seen with surgical drainage in addition to treatments for exposure keratopathy and amblyopia. These resulted in reduced intraocular pressure and improved visual acuities.* Conclusion*. There appears to be a difference in the overall management of PCG and DSCH between adults and children. A high index of suspicion as well as emergency surgical treatment for DSCH and associated conditions should be performed on pediatric patients that present with these challenges.

## 1. Introduction

Delayed Suprachoroidal Hemorrhage (DSCH) is considered a rare event after trabeculotomy combined with trabeculectomy and 5-Fluorouracil (combined TT + 5FU) application in the management of Primary Congenital Glaucoma in children. It is most often a visually debilitating complication of intraocular surgery. However, various studies have shown that vision can be restored with proper management [1]. The report of a pediatric patient with successful surgical intervention and visual restoration is presented.

## 2. Case Presentation

A documented informed consent was obtained from the patient to publish this case report.

**Figure 1:**
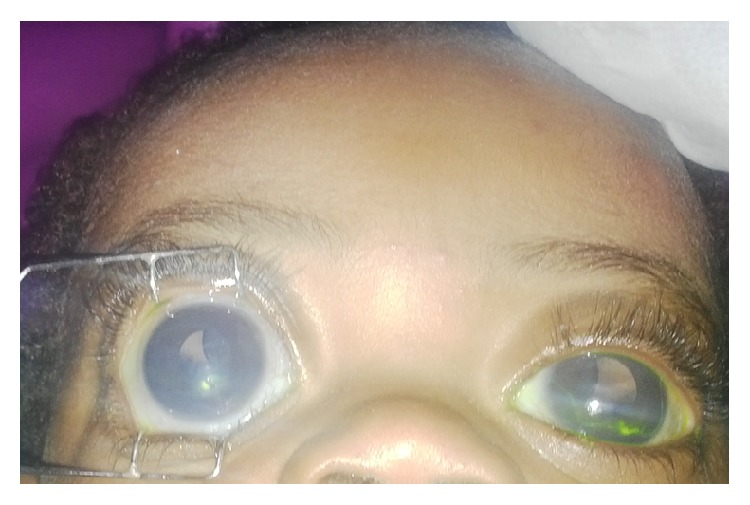
Child with bilateral buphthalmous and subluxated lenses.

An 8-month-old male child was first seen in the University of Calabar Teaching Hospital Department of Ophthalmology triage service on 26 March 2015, with complains of progressive bulging eyes from birth which was followed by bluish discoloration of the eyeball, and tearing and photophobia seen in both eyes (Figure 1). There was no history of consanguinity or family history of glaucoma or blindness. The child had used antiglaucoma eye drops given to him briefly from his referring ophthalmologist. There were no systemic associations.

The diagnosis of bilateral Primary Congenital Glaucoma (PCG) complicated with exposure keratopathy and ectopia lentis was made on the first day of presentation in clinic, based on the history and examination; see Table 1. The child was immediately started on Gutt Timolol 0.05% 12 hourly; Gutt Moxifloxacin 12 hourly, and Hypotears gel until surgery could be performed.

Examination Under Anaesthesia (EUA) and Combined Trabeculotomy Trabeculectomy and 5FU was performed as an emergency. Trabeculotomy was performed using the Harms Trabeculotome in a 180° degree fashion in both eyes left and right, respectively. Trabeculectomy was performed using the Moorfields Safer Surgery Surgical Technique [2] without the use of releasable sutures and an anterior chamber maintainer. Surgery was performed within 24 hours of presentation.

**Figure 2:**
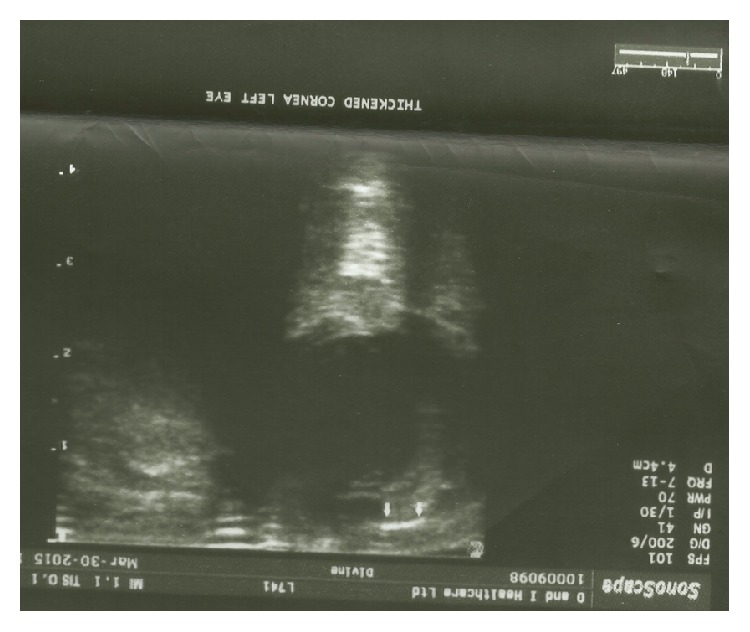
Left B ocular scan day #1 after operation showing suprachoroidal hemorrhage.

First day postoperative examination result is seen in Table 2. The patient was started on the following: Gutt Dexamethasone 2 hourly in the left and 4 hourly on the right, Occ Dexamethasone 12 hourly, Gutt Moxifloxacin 4 hourly, and Gutt Atropine 5% 8 hourly and Gutt Timolol 5% was discontinued. An hour after examination, the team was called back because the mother complained that she suddenly noticed blood in the left eye. There was no history of trauma.  Table 3 shows the result of examination of the left eye. The vision was no longer equal as the right eye was preferred. An urgent ocular B scan on 30 March 2015 was performed after 48 hrs as reported in Figure 2. It showed a dome-shaped choroidal detachment with an echolucent suprachoroidal effusion (likely hemorrhage). The child was booked for emergency EUA and drainage of suprachoroidal effusion on the same day. Please see Table 4 for surgical details.

Postoperative day one, following suprachoroidal drainage showed a deep anterior chamber with a serous fluid level of about 5%. The child was discharged home on the second day, 1 April 2015 after drainage, on the same medications including systemic prednisolone 5 mg daily for 6 weeks to come for follow-up in six weeks. The percentage packed cell volume was normal at discharge.

## 3. Follow-Up

**Figure 3:**
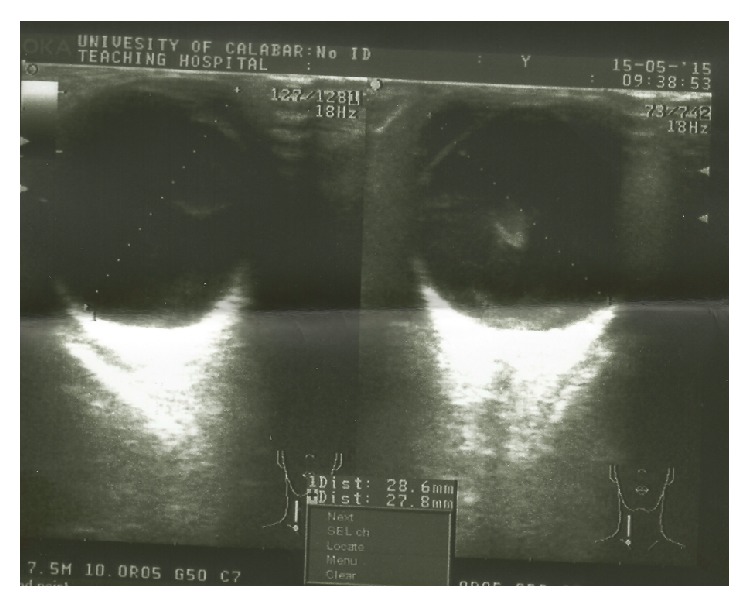
Left ocular B scan six weeks after suprachoroidal hemorrhage drainage.

A repeat ocular B scan 6 weeks later showed resolution of the suprachoroidal hemorrhage however with a residual clump of vitreous hemorrhage. Refer to Figure 3. Examination details in the follow-up clinic 6 weeks later are seen in Table 5. Right subconjunctival Gentamicin 20 mg and right temporary tarsorrhaphy were performed after the EUA for worsening right corneal epithelial loss from exposure keratopathy and for left amblyopia at this time. The child continued on all topical medications in the left eye. The tarsorrhaphy sutures were removed after 10 days. This was followed with patching of the left eye for 1 hour a week for one week. Visual acuity (VA) improved to 6/24 (Table 6). Patching was continued for one more week but the VA remained at 6/24 in that left eye. Maintenance patch of alternate day for 30 minutes for 4 weeks showed no change in VA.

## 4. Summary of Eye Pathologies

The child had various diagnoses at different phases in the management; they included bilateral Primary Congenital Glaucoma with poor intraocular pressure control on a single medication; Bilateral Ectopia Lentis; Right Exposure Keratopathy; Left Delayed Suprachoroidal Hemorrhage; left amblyopia; bilateral PCG with well-controlled IOP following combined TT + 5FU.

## 5. Summary of the Child's Visual Status on Presentation

The child presented as a severely visually impaired child and at discharge was mildly visually impaired.

## 6. Discussion

Surgeons treat Primary Congenital Glaucoma most commonly with goniotomy, trabeculotomy, trabeculectomy, combined trabeculotomy and trabeculectomy, tube shunt surgery, and cyclodestruction depending on the surgical skill, severity of the disease, and available technology [1–4]. However, the consensus today for the treatment of Primary Congenital Glaucoma is angle surgery in combination with trabeculectomy with antimetabolite use [2, 5]. It is a safe and effective procedure for primary congenital glaucoma, has a greater long-term success rate of up to 95% [2–4], and offers the best hope of success in advanced cases. It results in improvement in reduction of intraocular pressure as well as improvement in corneal clarity and reversal of the optic nerve abnormal morphology and the scleral coat, ultimately leading to an improvement in the visual status of the child. Trabeculectomy is currently performed with adjuvant use of antimetabolites such as mitomycin C or 5-Fluorouracil to enhance successful and prolonged functioning of the bleb. In spite of these advantages, trabeculectomy in adults and children is not without potential complications, even in the immediate postoperative period. The challenges of trabeculotomy include identification of Schlemm's canal through a distorted anatomy in most buphthalmic eyes and the difficulty of performing a 360° intervention on Schlemm's canal without the use of fibre or microcatheters or visual guidance.

We report our first case of suprachoroidal hemorrhage in a pediatric ophthalmology and strabismus unit, even though DSCH is reported to be a known complication following glaucoma surgery [1].

Delayed suprachoroidal hemorrhage (DSCH) was diagnosed clinically at the first day after operation, by the presence of a dark mass pushing the retina forward, with blood breaking through into the vitreous cavity and anterior chamber after combined trabeculectomy trabeculectomy with adjuvant 5FU therapy. Ultrasound B scan was used to confirm the diagnosis in this case.

Delayed suprachoroidal hemorrhage (DSCH) could occur hours or days after glaucoma surgery. The usual onset is sudden, with severe pain and loss of vision in adults [4]. In preverbal children this would present as irritability which is not unusual after any surgery in children, hence the importance of detailed ocular examination in making a clinical diagnosis in children.

One of the risk factors for the development of DSCH is an hypotonous eye. Further risk may be a Valsalva manoeuvre including coughing and straining, in a restless patient recovering from anesthesia. The usual high aphakic refractive error was overwhelmed by low hyperopia as a result of elongation of the globe. Aphakia is a noted risk factor in previous studies [6]. Our patient however was not aphakic but had an extensive subluxated lens. Aphakia may reduce the resistance to fluid accumulation in the suprachoroidal space [1]. Such suprachoroidal effusions are common after glaucoma operations in general. This is said to be a prerequisite to develop delayed suprachoroidal hemorrhage. In the phakic eye, the intact lens-iris diaphragm and vitreous resist scleral deformation during hypotonia, while in the aphakic eye, scleral folding may place added stress on the ciliary arteries.

In the management of suprachoroidal hemorrhage, instead of the usual 7-to-14-day observation period for clot lysis in adults, the hemorrhage was drained from the suprachoroidal space immediately after confirmed diagnosis and within 48 hours. The literature shows that eyes that have had drainage of the hemorrhage with reformation of the anterior chamber regained preoperative visual acuity [7, 8]. The duration from the onset of hemorrhage to drainage, in cases with successful outcomes, is reported to be within a week, with a range of 6–20 days [3]. However, the best timing and clear indications for the surgical drainage of the SCH is still undetermined in children. The time of intervention may be an important factor in the eventual visual outcome in children. We report a performance of drainage with 48 hrs of onset of the complication. This procedure was performed because of the threat of the onset of early corneal blood staining and amblyopia which are major causes of preventable visual loss in children. Loss of vision in this patient was from corneal edema and scarring, exposure keratopathy, optic nerve atrophy, ectopia lentis, and refractive error and amblyopia. These complications are commonly seen in children with late presentation of PCG.

Even though we have recorded an improvement in the vision and a reduction of the intraocular pressures, several challenges lie ahead in the management of this child. These include

1. What surgical procedure would be best to further bring down the pressure in both eyes, as an 8-week follow-up pressure of 21 and 22 mmHg may be an early indication of the risk of poor IOP control in future? The pressure rise may be contributed by the subluxated lenses which results from rise in intraocular pressure and accompanying growth of the eye ball which ends in the zonules of the lens becoming stretched. Considering the current available surgical interventions, would glaucoma valve or stent surgery be the ideal surgery to perform considering that there may be a need for repeat procedures and with each additional surgical intervention there is a consequent visual compromise or would a Cyclophotoabaltion procedure be preferable in titrated doses in this case?
2. Should the subluxated lenses be interfered with now or later? If later when and what factors would guide such a surgical decision? Further, what type of surgical procedure should be performed for the subluxated lenses? Would the preferred surgical technique be lensectomy with anterior vitrectomy or a vectis extraction with anterior vitrectomy?
3. What refractive treatment would be best for this preverbal child? If intraocular surgery to the lens is performed, how will the refractive error be corrected? Would it be best to leave the child aphakic or pseudophakic?

## 7. Lessons Learnt

From this case we are reminded that tearing and corneal opacity in a child with buphthalmous from PCG can be due to other causes, to be prepared to manage exposure keratopathy in a child with PCG, DSCH can occur after uneventful intraocular combined TT + 5FU surgery for PCG in children, DSCH is an emergency in children, different from adults where intervention can be delayed for up to 14 days, when there is a significant difference in IOP between two simultaneous surgically treated eyes with combined TT + 5FU, there should be a suspicion of DSCH in the eye with the higher pressure, and lastly vision should be monitored in each eye and to watch out for amblyopia.

## 8. Conclusion

DSCH can occur as a postoperative complication in children who have subluxated, buphthalmic eyes from PCG and who have had combined TT + 5FU. This child with DSCH had a good immediate visual prognosis in the affected eye. It appears that such a result may be due partly to early recognition of the surgical complication and the immediate surgical and amblyopia interventions.

## Figures and Tables

**Table 1:** Findings at first presentation in the children's clinic.

OD	Examination	OS
<6/60	VA (CSM method unaided)	<6/60

+6.00 − 3.00 Å~117 degrees	Objective refraction (aphakic portion)	+6.00 − 2.50 × 98 degrees

30.36 mm	Axial length (contact method)	27.65 mm

50 mmHg	IOP (ICARE)	54 mmHg

Blue	Sclera	Blue

V: 17 mm H: 17 mm	Corneal diameter	V: 17 mm H: 16 mm
Fluorescein epithelial stain +ve, inferiorly about 1 mm Exposure keratopathy Haab striae, hazy cornea Stromal edema	Cornea	Fluorescein epithelial stain −ve with stained mucoid strand Haab striae, hazy cornea

Deep	Anterior chamber	Deep

5 mm, unreactive	Pupil	5 mm, unreactive

Superonasal subluxation	Lens	Superonasal subluxation

Clear	Vitreous	Clear

C/D 0.8 Distinct margin Irregular neuroretinal rim, pale, cupped disc, eccentric cup Normal caliber vessels, Nasalization of vessel Normal retina	Fundi exam with +28D and +20	C/D 0.85 Distinct margin Irregular neuroretinal rim, pale, cupped disc, eccentric cup Normal caliber vessels Nasalization of vessel Normal retina

**Table 2:** First Postoperative day (24 hours after surgery). Initial eye drops of Gutt Tetracaine instilled × 3 to prepare the eye for examination. The child was examined in the children's eye ward.

OD	Examination	OS
6/60	VA (CSM method)	6/60

5 mmHg	IOP (ICARE)	14 mmHg

Blue	Sclera	Blue

Fluorescein stain +ve, inferiorly Clearer cornea centrally and superiorly, Fl +ve 1 mm	Corneal	Fluorescein stain −ve Clearer cornea

Deep, no residual hyphema	Anterior chamber	Deep, no residual hyphema

5 mm, unreactive	Pupil	5 mm, unreactive

Superonasal subluxation	Lens	Superonasal subluxation

Clear	Vitreous	Clear

Bright red reflex, disc seen	Fundi exam with +28D	Bright red reflex, disc seen

**Table 3:** Results of EUA performed 48 hrs after surgery.

OD	Examination	OS
6 mmHg	IOP (ICARE)	46 mmHg
	Sclera	Tight scleral flap
Flu +ve 1.5 mm, inferiorly	Corneal	Edema, blood stain centrally
	Anterior chamber	Deep, hyphema + serous fluid 70%
	Pupil	7 mm, unreactive
	Lens	Superonasal subluxation
	Vitreous	Dull red reflex, hemorrhage
	Fundi exam with +28D	No details
	Refraction	No reflex

**Table 4:** Surgical intervention to the left eye.

Anatomic site and surgical intervention	OS
Conjunctiva	Conjunctival sutures released and closed at the end of the procedure

Sclera (trabeculectomy site)	Left and right outer flap suture released. Inner ostium examined, small blood clot of 1 mm removed by AC irrigation Irrigation flow unobstructed, satisfactory flow rate Single loose suture to outer scleral flap centrally placed

Anterior chamber	AC paracentesis at 5.30, irrigation of AC with outward flow through inner scleral ostium, air left in AC AC deep with no collection

Sclera	Extrascleral drainage with radial and deep sclerotomies laterally, effluent of about 20 mLs altered blood and serous removed with gentle pressure on the globe, until there is no more effluent, sclerotomy closed

**Table 5:** Examination under anesthesia six weeks after delayed suprachoroidal hemorrhage drainage.

History	Improved vision with clearing of the white patch in both eyes, eyes look smaller than before surgery, tearing and blepharospasm eye blepharospasm in right eye	

OD	Examination	OS

6/24	CSM unaided	6/60

Buphthalmos less Lagophthalmos++	Globe	Buphthalmos less

Low diffuse blebs	Conjunctiva	Low diffuse blebs

Corneal clear centrally, stromal scars with exposure keratopathy, fluorescein +ve measuring 3 mm inferiorly	Cornea Corneal diameter	Corneal clear with stromal scars Fluorescein −ve

18 mmHg	IOP ICARE	22 mmHg

Normal, brown	Iris	Normal, brown
Superomedial subluxation, clear	Lens	Superomedial subluxation, clear

	Vitreous	A clump of vitreous hemorrhage inferiorly quadrant about 3 mm

C/DR = 0.7	Fundoscopy/BIO	Pink oval disc C/DR = 0.8 Moderate chorioretinal degeneration, flat retina

+4.50DS/−2.00DC × 170°	Objective refraction (through the aphakic portion)	+4.00DS/−2.50DC × 95°

**Table 6:** Eight weeks after combined trabeculotomy + trabeculectomy 5FU and left delayed suprachoroidal drainage in clinic.

History	Improved vision with clearing of the white patch in both eyes, eyes looking smaller than before surgery, no tearing, no blepharospasm	

OD	Examination	OS

Showing axial lengths of 28.6 mm	B scan	Axial lengths of 26.0 mm, no other pathology seen except increased vitreous echogenic strands

6/18	VA CSM aided with spectacles	6/24

Buphthalmos less	Globe	Buphthalmos less

Low diffuse blebs	Conjunctiva	Low diffuse blebs

Corneal clear centrally, stromal scars fluorescein −ve	Cornea	Corneal clear with stromal scars

21 mmHg	IOP ICARE	22 mmHg

Normal, brown	Iris	Normal, brown
Superomedial subluxation, clear	Lens	Superomedial subluxation, clear

Clear	Vitreous	No proliferative vitreoretinopathy

C/DR = 0.7	Fundoscopy/BIO	Pink oval disc v.C/DR = 0.8 Moderate chorioretinal degeneration No retinal detachment

+4.50DS/−2.00DC × 90°	Objective refraction (through the aphakic portion)	+4.00DS/−2.50DC × 85°
